# Ectopic expression of TWIST1 upregulates the stemness marker OCT4 in the esophageal squamous cell carcinoma cell line KYSE30

**DOI:** 10.1186/s11658-017-0065-x

**Published:** 2017-12-29

**Authors:** Mohammad Hossein Izadpanah, Mohammad Reza Abbaszadegan, Yasaman Fahim, Mohammad Mahdi Forghanifard

**Affiliations:** 1 Division of Human Genetics, Immunology Research Center, Avicenna Research Institute, Mashhad University of Medical Sciences, Mashhad, Iran; 2 Department of Biology, Damghan Branch, Islamic Azad University, P.O.Box: 3671639998, Cheshmeh-Ali Boulevard, Sa’dei square, Damghan, Islamic Republic of Iran

**Keywords:** Esophageal squamous cell carcinoma (ESCC), Cancer stem cells (CSCs), *TWIST1*, *OCT4*

## Abstract

**Background:**

The transcription factor TWIST1 plays an important role in the epithelial–mesenchymal transition (EMT) process and in the migration, invasion and metastasis of cancer cells. OCT4, which is a homeobox transcription factor, has an important role in the self-renewal potential of cancer cells. Our aim here is to elucidate impact of ectopic expression of TWIST1 on OCT4 gene expression in esophageal squamous cell carcinoma (ESCC).

**Methods:**

The ESCC line was KYSE30. GP293T cells were transfected with purf-IRES-GFP and pGP plasmids to produce recombinant viral particles. A semi-confluent KYSE30 culture was transduced with the prepared retroviral particles. mRNA extraction and cDNA synthesis were performed from normal KYSE30 cells and those ectopically expressing *TWIST1*. Expressional analysis of *TWIST1* and *OCT4* were performed with relative comparative real-time PCR.

**Results:**

Ectopic expression of *TWIST1* in KYSE30 cells was related to its significant overexpression: nearly nine-fold higher in GFP-h*TWIST1* KYSE-30 cells than in control GFP cells. This induced expression of *TWIST1* caused significant upregulation of *OCT4* in GFP-h*TWIST1* KYSE-30 cells: nearly eight-fold higher. In silico analysis predicted the correlation of *TWIST1* and *OCT4* through *ETS2*.

**Conclusions:**

Overexpressed *TWIST1* can be correlated with upregulation of the cancer stem cell marker *OCT4* and the protein may play critical regulatory role in *OCT4* gene expression. Since OCT4 is involved in the self-renewal process, the results may suggest a new linkage between *TWIST1* and *OCT4* in the cell biology of ESCC, highlighting the probable role of TWIST1 in inducing self-renewal.

## Introduction

Esophageal cancer (EC) is the eighth most common cancer and the sixth leading cause of cancer-related death worldwide [1]. Geographical factors, local culture and ethnicity have an influence on the incidence rate of EC in different regions [2].

Esophageal squamous cell carcinoma (ESCC) is the sixth most common cancer among men and ninth among women worldwide. Although surgery is therapeutically useful in the early stages of ESCC, most patients are diagnosed in the late stages of the disease, when common therapeutic methods, including surgery, chemotherapy and radiotherapy, are not effective enough to inhibit recurrence. Therefore, a more effective targeted therapy is needed to increase ESCC patient survival [3].

TWIST1 belongs to a class of transcription regulators with a basic helix-loop-helix (bHLH) DNA-binding domain. It identifies a hexanucleotide consensus sequence called E-box (CANNTG) in the promoter region of target genes [4]. Having recognized the E-boxes, TWIST1 can regulate downstream gene expression.

TWIST1 is also known to be involved in the complex process of epithelial–mesenchymal transition (EMT), which plays a role in the migration of cells during their development. EMT is also believed to have an important role in tumor invasion and metastasis, with *TWIST1* upregulation enhancing this ability in different types of cancer cells [5, 6] including melanoma, nasopharyngeal carcinoma, ESCC, and breast, uterine, prostate, pancreatic, gastric and cervical cancers [7–9]. *TWIST1* overexpressing cells show a significantly elevated level of cancer stem cell-like traits, such as tumorsphere formation, ALDH1 and CD44 gene expression, and activated β-catenin and Akt pathways [10].

OCT4 belongs to the POU domain family, the members of which play an important role during embryonic development. It is a multifunctional factor involved in stem cell self-renewal and differentiation, and in carcinogenesis [11]. *OCT4* expression has been proved in mouse and human inner cell mass (ICM) cells, embryonic stem cells, germ cells, embryonic carcinoma cells and embryonic germ cells (pluripotent cells). It is activated mostly in undifferentiated stem cells [12, 13].

As the main stemness state marker, *OCT4* is expressed in over 93% of ESCCs [14]. The expressions of stem cell markers such as *OCT4* and survivin are closely related to the surgical stages of the disease and correlate with poor survival of patients. Since *OCT4*- or survivin-positive tumors are associated with much poorer prognosis than *OCT4*- or survivin-negative tumors [15], there may be a correlation between these stem cell markers and other markers of poor prognosis in ESCC, such as TWIST1.

Our aim was to evaluate the effect of *TWIST1* upregulation on *OCT4* gene expression in an ESCC cell line, KYSE30, and to evaluate a probable new route relating the contributions of these two important genes to ESCC development.

## Materials and methods

### In silico sequence analysis

The mRNA and gene sequences of *OCT4* were obtained from Genebank (accession numbers NM_203289 and NC_000006.12, respectively). The sequence analysis was conducted using CLC Main Workbench version 7 (CLC bio).

### Cell lines and culture conditions

KYSE-30 and GP293T cell lines were purchased from the Pasteur Institute and respectively cultured in RPMI1640 or DMEM containing 10% fetal bovine serum (FBS) and 1% pen-strep at 37 °C in a 95% humidity atmosphere with 5% CO_2_.

### Retroviral transduction and overexpression study

The GP293T cell line was transfected with 5 μg of plasmid purf-IRES-GFP (pruf-IRES-GFP-h*TWIST1*) and 4 μg of pGP plasmid in 500 ml of DMEM without supplements, using X-tremeGENE HP DNA reagent (Roche Diagnostics GmbH), as described previously [16, 17]. Infectious particles were harvested from the supernatant and filtered through a 0.45-μm Nalgene filter (Nalgene Labware).

A semi-confluent KYSE-30 culture (1 × 10^5^ cells/6-well in RPMI-60 + 10% FBS) was transduced with prepared recombinant retroviral particles. To determine the transfection accuracy, inverted fluorescence microscopy (Olympus IX-70) was used to observe stably transduced highly expressing GFP (control) and GFP-h*TWIST1* KYSE-30 cells (>95% positive).

### Comparative real-time PCR

Total RNA was extracted from 3 × 10^3^ of both GFP-hTWIST and GFP-control KYSE-30 cells and transduced KYSE-30 cells using an RNeasy Mini Kit (QIAGEN). After cDNA synthesis using an Easy cDNA Reverse Transcription Kit (Fermentas), relative comparative real-time PCR of *TWIST1* and *OCT4* mRNA expression was performed using SYBR green PCR Master Mix (Fermentas) on a Stratagene Mx-3000P Real-Time Thermocycler (Stratagene) with the primer sets shown in Table 1. The thermal profile was 10 min at 95 °C followed by 40 cycles of 15 s at 95 °C, 30 s at 57 °C, and 45 s at 72 °C. Glyceraldehyde 3-phosphate dehydrogenase (GAPDH) was used to normalize gene expression, and PCR efficiencies for *GAPDH* and *OCT4* were verified by generating standard curves [18].

**Table 1:** Primer sequences used in real-time PCR

	Forward primer	Reverse primer
*TWIST1*	GGAGTCCGCAGTCTTACGAG	TCTGGAGGACCTGGTAGAGG
*OCT4*	CCTGAAGCAGAAGACGATCA	CCGCAGCTTACACATGTTCT
*GAPDH*	GGAAGGTGAAGGTCGGAGTCA	GTCATTGATGGCAACAATATCCAC

### Prediction of gene–gene interaction

The probable predictive connection between *OCT4* and *TWIST1*, including annotation, cotreatment expression and protein–protein interaction was obtained using the *biograph* database (http://biograph.be/).

### Statistical analysis

Data analyses were performed using the SPSS 19.9 statistical package. The correlation between gene expressions was assessed using either the χ2 or Fisher exact tests and Pearson’s correlation. *p* < 0.05 was considered statistically significant.

## Results

### Sequence analysis of *OCT4* promoter

**Fig. 1:**
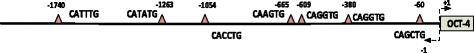
A schematic view of the positions and sequences of seven E-box hexanucleotide consensus sequence CANNTG within 2 Kb upstream of the *OCT4* transcription start site

The sequences of the *OCT4* transcription unit and its upstream region were examined for the existence of probable E-boxes. Seven different E-boxes were found in a 2-kb region upstream of the *OCT4* transcription start site. Interestingly, two of the E-boxes were located close to the transcriptions start site, in positions −60 and −380. Other E-boxes were scattered from −609 to −1740 (Fig. 1). Furthermore, there were 85 E-boxes in the *OCT4* transcription unit, of which 20 were located in exonic regions, while the remainder were distributed in the introns (Table 2).

**Table 2:** The number and positions of E-box hexanucleotide consensus sequence (CANNTG) in OCT4 transcription unit

Sequence	Number	Positions
CACTTG	8	506–511, 4882–4887, 5233–5238, 5573–5578, 8676–8681, 12,560–12,565, 13,578–13,583, 15,856–15,861
CAGGTG	13	1160–1165,1527–1532,2430–2435,2951–2956^*^,3435–3440,3773–3778,4067–4072,6173–6178,10,155–10,160^*^, 10,695–10,700,14,196–14,201^*^,14,380–14,385^*^,15,862–15,867^*^
CAAGTG	8	1283–1288,1518–1523,10,518–10,523,11,353–11,358,13,240–13,245,13,593–13,598^*^,13,613–13,618^*^,14,154–14,159^*^
CATCTG	2	2013–2018,15,053–15,058
CAGCTG	11	2180–2185,2951–2956^*,^4829–4834,5708–5713^,^6782–6787^*^,7763–7768^,^7939–7944,9308–9313,9488–9493,10,593–10,598,10,635–10,640
CACCTG	15	2239–2244,3538–3543,3926–3921,4010–4015,4998–5003,6898–6903^*,^6922–6927^*^,9443–9448,10,121–10,126^*^,10,587–10,529,12,259–12,264,12,518–12,523,13,823–13,828^*^,15,348–15,353,15,435–15,440
CATTTG	4	2306–2311,4433–4438,11,264–11,269,14,514–14,519^*^
CATATG	3	2538–2543,6233–6238,11,855–11,860
CAGATG	5	2556–2561,5599–5604,5700–5705,9206–9211,11,951–11,956
CAGTTG	4	4390–4395,5737–5742,9884–9889,12,014–12,019
CAAATG	1	4397–4402
CACATG	2	4569–4574,5850–5855
CAACTG	6	4850–4855,6768–6773^*,^10,665–10,670,10,728–10,733,15,680–15,685,16,283–16,288^*^
CATGTG	2	5057–5062,15,092–15,097
CAATTG	1	11,487–11,492*

The asterisks indicate exonic E-boxes

### Forced expression of *TWIST1* upregulated *OCT4* expression

**Fig. 2:**
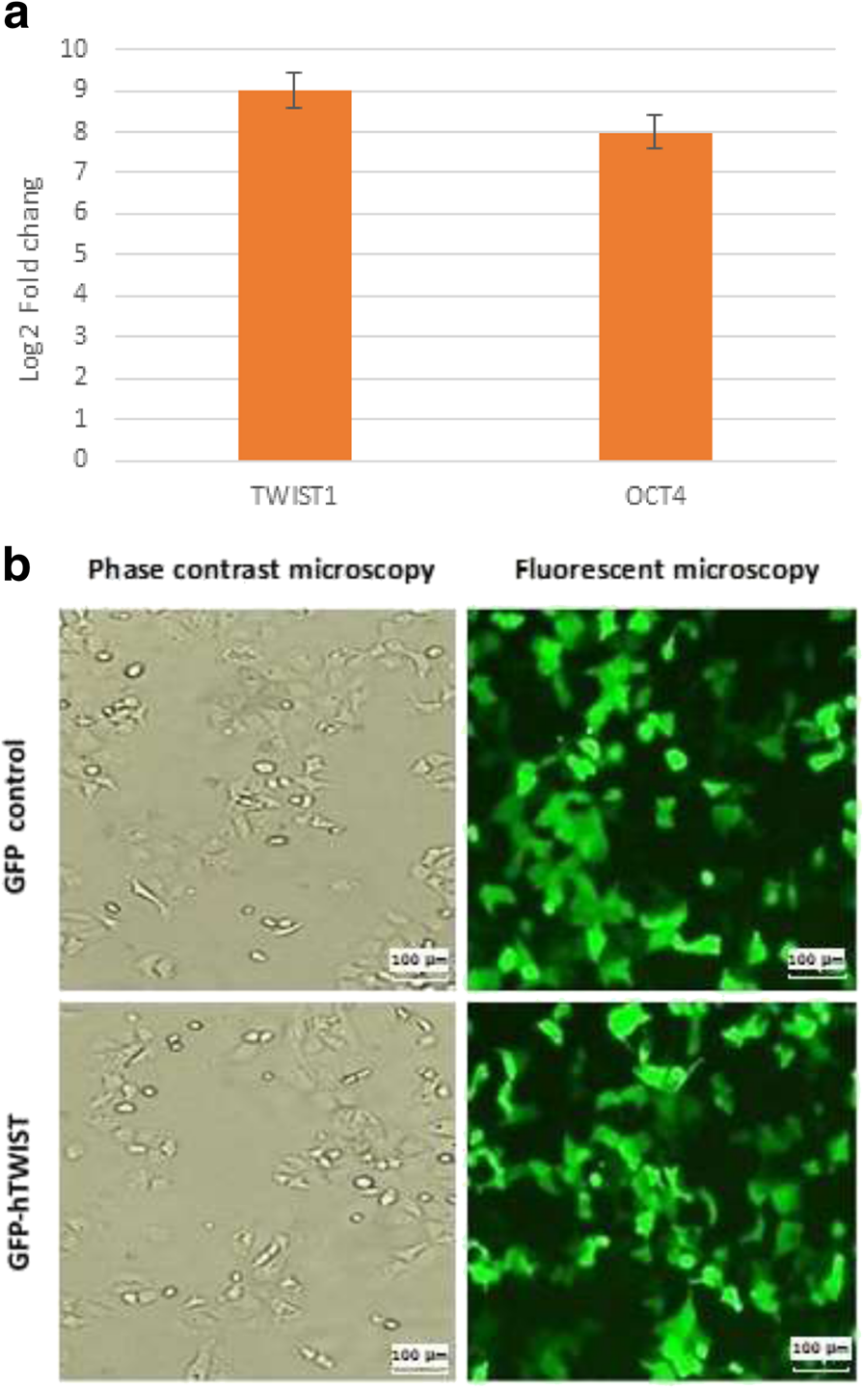
Enforced expression of *TWIST1* upregulates *OCT4* mRNA expression in KYSE30 cells. **a** – *TWIST1* is overexpressed nine-fold in GFP-hTWIST1 compared to the control. This caused a statistically significant eight-fold increase in *OCT4* mRNA expression. **b** – Phase contrast and fluorescent microscope images of GFP-hTWIST1 and control cells. The phase contrast images show the cells after 24 h and the fluorescent images are after 48 h

After the transduction of KYSE30 cells, a functional study was performed to evaluate the expression of *TWIST1* and *OCT4*. *TWIST1* was overexpressed nearly nine-fold in GFP-h*TWIST1* KYSE30 cells compared to the control. This level of *TWIST1* overexpression caused a significant increase in *OCT4* gene expression: nearly eight-fold in GFP-h*TWIST1* KYSE30 cells compared to the control (Fig. 2a). The phase contrast and fluorescent microscopy images of GFP-h*TWIST1* and GFP control KYSE30 cells are shown in Fig. 2b.

### TWIST1 and OCT4 may be linked through the ETS2 gene

**Fig. 3:**
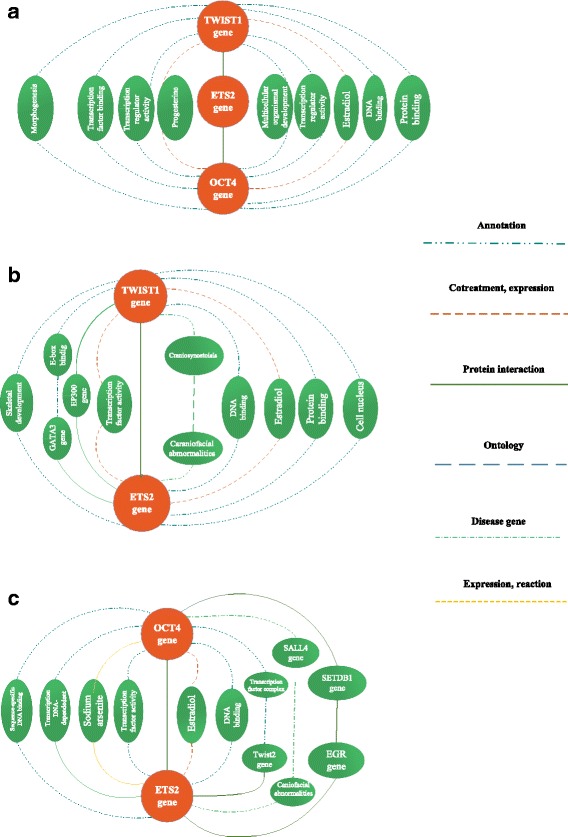
Computational relationship between genes based on the *biograph* database: http://www.biograph.be. **a** – Computational relationship between the TWIST1 and OCT4 genes. **b** – Computational relationship between the TWIST1 and ETS2 genes. **c** – Computational relationship between the OCT4 and ETS2 genes

Having checked the probable interactions of TWIST1 and OCT4, we found that ETS2 can act as an intermediate communicator for these markers (Fig. 3a). TWIST1 can indirectly interact with OCT4 through ETS2 via protein–protein interaction. There is also predicted evidence for indirect interaction of these genes in different cellular processes, such as morphogenesis and regulation of gene transcription, through their DNA- or transcription factor-binding activities.

## Discussion

As a bHLH transcription factor, TWIST1 effectively plays a role in EMT, with a controlling function in the migration, invasion and metastasis of cancer cells [19]. Its overexpression has been reported in a variety of invasive tumors [20–30]. Such an elevated level of *TWIST1* expression may have significant impact on the cellular transcription network and change cell behavior through deregulation of different cell signaling pathways [31].

In this study, *TWIST1* was ectopically expressed in KYSE30 cells and its effect on the transcription of the stemness state marker *OCT4* was investigated. Interestingly, *TWIST1*-transduced cells showed higher levels of *OCT4* expression than the GFP control cells. This may both highlight the impact of *TWIST1* on *OCT4* expression and introduce a novel link between *TWIST1* and the stemness state of cancer cells.

The crosstalk between *TWIST1* and intermediaries of different cell signaling pathways has previously been demonstrated. It has been reported that *TWIST1* has an inverse correlation with *SNAIL* in ESCC KYSE30 cells, with the suggestion that enforced expression of *TWIST1* may negatively regulate *SNAIL* expression [16]. Furthermore, a new connection between *TWIST1* and the testicular cancer antigen *MAGEA4* was recently reported for KYSE30 cells. The performed functional study on ESCC cells showed an elevated level of *MAGEA4* expression after *TWIST1* ectopic expression, and confirmed indirect binding of *TWIST1* to the *MAGEA4* promoter region leading to increased expression of *MAGEA4* at both the mRNA and protein levels [17].

In addition, a significant correlation between *TWIST1* and *MAML1*, the main transcription factor of the Notch signaling pathway, was reported for ESCC patients through advanced stages of the disease, suggesting new crosstalk between these markers in ESCC invasion and metastasis [32].

This evidence clearly shows the great potential of TWIST1 in transcription regulation of a wide spectrum of genes involved in various cell signaling pathways. In line with this statement, the impact of *TWIST1* ectopic expression on *OCT4* gene expression was revealed in this study. The *OCT4* upstream region consists of a proximal promoter located near the transcription start site and a number of distal enhancers. As shown in Fig. 2, there are seven different E-boxes in the *OCT4* promoter region. *TWIST1* may bind either directly or indirectly to these sequences and transcriptionally upregulate *OCT4* gene expression. Confirming this correlation, our in silico analysis introduced ETS2 protein as an important linkage between TWIST1 and OCT4. As depicted in Fig. 3, TWIST1 and OCT4 can be associated with ETS2 through protein–protein interactions. Figure 3b shows some predicted paths of connection between TWIST1 and ETS2.

ETS is a downstream target of ERK1*/2* and one the mitogen-activated protein kinase-dependent transcription factors, which can interact with coregulatory transcription factors, including bHLH transcription factors such as TWIST1. Based on its partner type, it can activate or repress the transcription of numerous target genes involved in cancer cell progression and invasion [33, 34].

The ability of TWIST1 to interact with ETS2 may be correlated to the capability of TWIST1 to repress Ras-mediated activation of p16INK4A in epithelial cancer cells [35]. An association between ETS2 and TWIST1 was also confirmed in *Helicobacter pylori*-infected gastric cancer cells (GCCs), where the two genes were found to enhance *SIAH2* expression [36].

OCT4 also contributed significantly to the progression of ESCC. It has been reported to positively regulate survivin expression, promoting cancer cell proliferation and leading to a poor prognosis and poor survival of ESCC patients [15]. Furthermore, a possible role of OCT4 in identifying putative cancer stem cells in ESCC pathobiology has been determined [37].

It has been revealed that OCT4 and ETS2 may be linked together. OCT4 can prevent the tendency of pluripotent cells to differentiate through its ability to repress the ETS2 activity. A soluble complex containing these proteins is recognized in fully pluripotent ES cells [38]. Figure 3c depicts predicted linkages between OCT4 and ETS2.

Here, we showed the correlation between *TWIST1* and *OCT4* and hypothesized the transcriptional regulation of *OCT4* by *TWIST1*. To confirm this statement, further experiments are required. For example, the generation of truncated mutants of the *OCT4* promoter region and luciferase assays are needed to determine which E-box sites (Fig. 1) are essential to TWIST1-mediated regulation of *OCT4* expression. Chromatin immunoprecipitation assays (CHiP) may help to elucidate the role of TWIST1 binding to the *OCT4* promoter. In addition, an electrophoretic mobility shift assay (EMSA) would determine the direct or indirect binding of TWIST1 to the *OCT4* promoter.

## Conclusions

We showed that ectopic expression of *TWIST1* in KYSE30 cells can upregulate *OCT4* gene expression at the mRNA level. Based on the existence of different E-boxes in the *OCT4* promoter region sequence, it may be hypothesized that TWIST1 binds either directly or indirectly to these and thus regulates *OCT4* gene expression.

To the best of our knowledge, this is the first report on the connection between *TWIST1* and *OCT4* to introduce a correlation between *TWIST1* and the stemness state in cells of the ESCC line KYSE30. Such linkage may expand our understanding of the biological role of *TWIST1* in cancer cell progression and self-renewal.

## Abbreviations

bHLH: Basic helix-loop-helix
cDNA: Complementary DNA
CSCs: Cancer stem cells
DMEM: Dulbecco’s Modified Eagle’s medium
EMT: Epithelial–mesenchymal transition
ESCC: Esophageal squamous cell carcinoma
ETS2: ETS proto-oncogene 2
FBS: Fetal bovine serum
GAPDH: Glyceraldehyde 3-phosphate dehydrogenase
GFP: Green fluorescent protein
ICM: Inner cell mass
OCT4: Octamer-binding transcription factor 4
